# Diffuse pigmented villonodular synovitis treated with arthroscopic total synovial peel

**DOI:** 10.1186/s12893-023-01906-x

**Published:** 2023-01-16

**Authors:** Hao-Qiang Song, Guo-Feng Wu, Wei-Zhong Qi, Li-Jun Lin

**Affiliations:** 1 Department of Joint and Orthopedics, Zhujiang Hospital, Southern Medical University, Guangzhou, Guangdong People’s Republic of China; 2 Department of Orthopedics, South University of Science and Technology Hospital, Shenzhen, China

**Keywords:** Pigmented villonodular synovitis, Arthroscopic total synovial peel, Relapse rates

## Abstract

**Background:**

Diffuse pigmented villonodular synovitis (PVNS) is prone to recurrence after surgery, and it is difficult to achieve a long-term complete cure.

**Objective:**

To reduce the recurrence rate of PVNS, the author pioneered the arthroscopic total synovial peel (ATSP).

**Methods:**

From March 2014 to July 2020, a total of 19 patients (6 males and 13 females) with diffuse PVNS of the knee were treated in our department and underwent ATSP. It’s ‘peel’ rather than simple excision. This method is similar to peeling bark. Relapse rates and functional scores were determined, with follow-ups ranging from 12 to 72 months, on average 36 months.

**Results:**

Treatment efficacy was assessed by imaging and functional scores. Imaging results indicated a recurrence rate of 10.5%. In patients without recurrence, the visual analog score (VAS) decreased from 4.76 ± 2.02 preoperatively to 1.56 ± 1.15 postoperatively. The Tegner-Lysholm knee function score (TLS) score increased from 67.76 ± 15.64 preoperatively to 90.32 ± 8.32 postoperatively. Compared with the literature, ATSP significantly reduces the postoperative recurrence rate of diffuse PVNS. The preliminarily findings suggest that this approach could greatly reduce the recurrence rate of postoperative PVNS in follow-up studies.

**Conclusion:**

This approach may be a viable option for treating diffuse PVNS via arthroscopy and is worthy of clinical consideration.

## Introduction

Diffuse pigmented villonodular synovitis (PVNS) is a group of chronic synovial proliferative lesions of unknown etiology which are inflammatory and benign, often occurring in joints, tendons, and synovium [1]. PVNS usually occurs in adults between the ages of 20 and 40 years and most often involves the synovium of large joints, especially the knee [2]. At present, the main treatment method is arthroscopic total synovectomy. Postoperative recurrence rates are high with either open or arthroscopic surgery, especially for diffuse PVNS of the knee joint, and repeated attacks of PVNS can lead to damage to articular cartilage. The destruction of bone affects joint function [3–5]. Therefore, the complete removal of the synovium of the lesions and the reduction of recurrence are the basic principles of diffuse PVNS treatment [6, 7]. To completely remove diffuse PVNS synovial lesions, the Department of Joint and Orthopedics of Zhu Jiang Hospital of Southern Medical University innovatively adopted the method of arthroscopic total synovial peel (ATSP). The purpose of this study was to test whether ATSP can effectively reduce the recurrence of diffuse PVNS and its effects on patients’ pain scores and Tegner Lysholm knee function scores.

## Materials and methods

### Study design

**Fig. 1:**
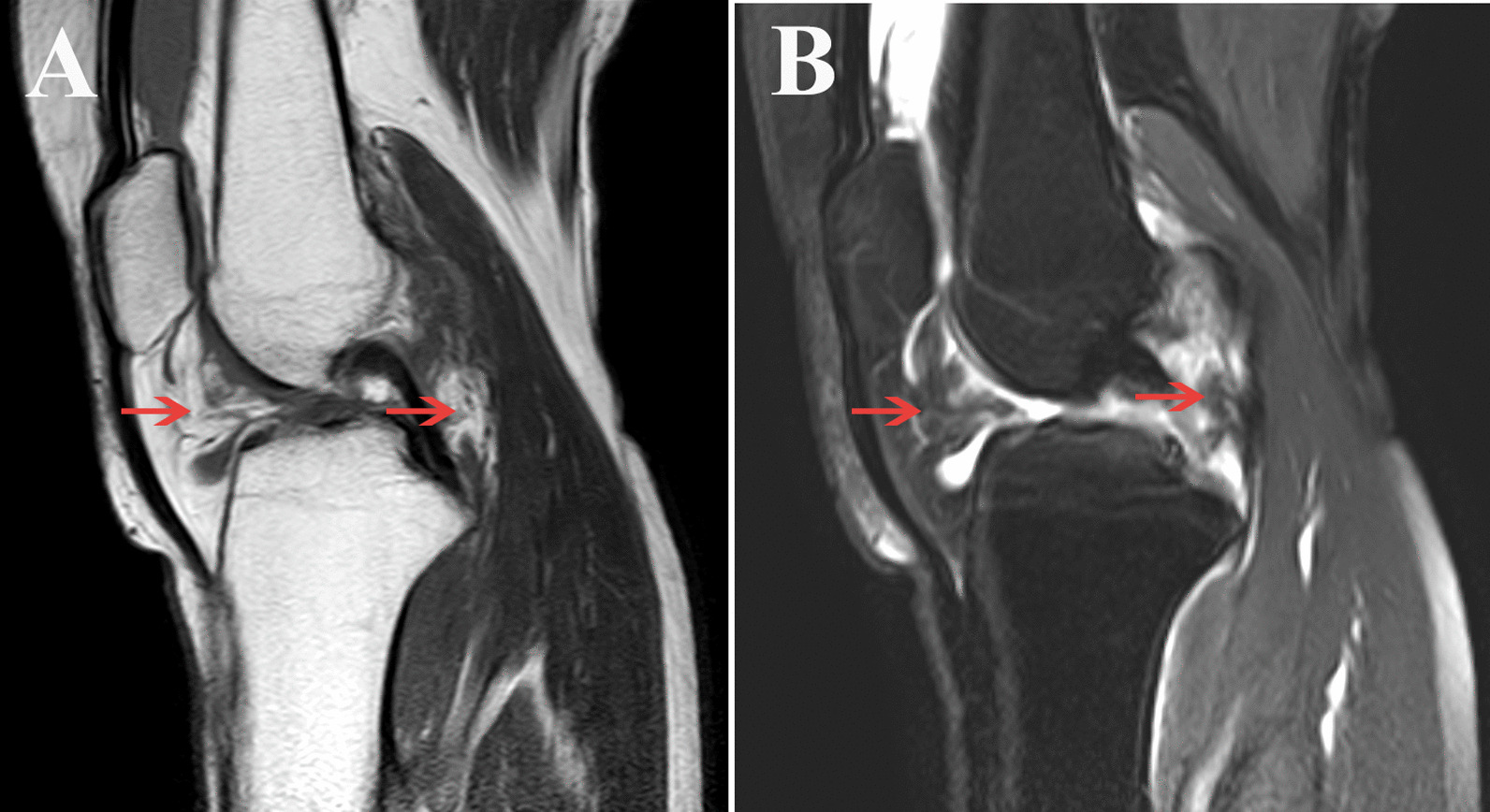
Preoperative MRI showed that the synovial membrane of the knee joint was significantly thickened, showing nodular low T2WI and T1WI low signal changes. **A**. T1WI, **B**. T2WI. Red arrows indicate mainly located in the infrapatellar fat pad and next to the cruciate ligament

From March 2014 to July 2020, a total of 19 patients (6 males and 13 females) with diffuse PVNS of the knee were treated in our department, aged 6–78 years (average 39.8 years), and the course of the disease was 20 days−10 years. This study have been performed in accordance with the Declaration of Helsinki and been approved by the ethics committees of Zhu Jiang Hospital of Southern Medical University ethically and provided the corresponding ethical certificate (No. 2021-KY-165-03) and informed consent. All patients complained of varying degrees of knee joint swelling, pain, and dysfunction before surgery. A preoperative MRI examination was performed for the initial diagnosis of diffuse PVNS as shown in Fig. 1, then all cases were confirmed to be PVNS by postoperative pathology. The postoperative follow-up time was 1–6 years. As shown in Table 1 below.

**Table 1:** Patient information

Number	Age (years)	Duration of symptoms (months)	Treatment	Pre-op VAS score	Post-op VAS score	Pre-op TLS score	Post-op TLS score	pathology	Recurrence	Follow-up (months)
1	20	5	ATSP	7	2	64	90	PVNS	No	72
2	38	48	ATSP	4	2	64	90	PVNS	No	70
3	36	1	ATSP	1	0	70	80	PVNS	No	69
4	78	120	ATSP	8	3	60	80	PVNS	Yes	67
5	45	108	ATSP	7	3	64	100	PVNS	No	57
6	30	6	ATSP	6	2	60	90	PVNS	No	62
7	49	12	ATSP	3	1	80	100	PVNS	No	39
8	50	4	ATSP	3	0	90	98	PVNS	No	39
9	8	10	ATSP	3	0	66	100	PVNS	No	31
10	6	48	ATSP	4	1	72	80	PVNS	No	31
11	27	6	ATSP	8	3	60	72	PVNS	Yes	25
12	58	24	ATSP	5	2	64	90	PVNS	No	25
13	64	5	ATSP	7	3	76	90	PVNS	No	25
14	67	24	ATSP	7	2	48	82	PVNS	No	23
15	52	6	ATSP	2	0	60	80	PVNS	No	22
16	16	2	ATSP	6	2	24	100	PVNS	No	21
17	50	12	ATSP	2	0	38	84	PVNS	No	16
18	41	24	ATSP	5	2	72	88	PVNS	No	12
19	23	12	ATSP	5	0	36	100	PVNS	No	13

*ATSP* arthroscopic total synovial, *PVNS* pigmented villonodular synovitis

### Main equipment

The 4.0 mm, 30 ° angle arthroscopy, cold light source, camera imaging system and planing system produced by Shrek company of the United States (model 1488-010-0001). The plasma cold ablation instrument and the electric pneumatic tourniquet produced by Smith & nephew company of the United Kingdom.

### Surgical methods

**Fig. 2:**
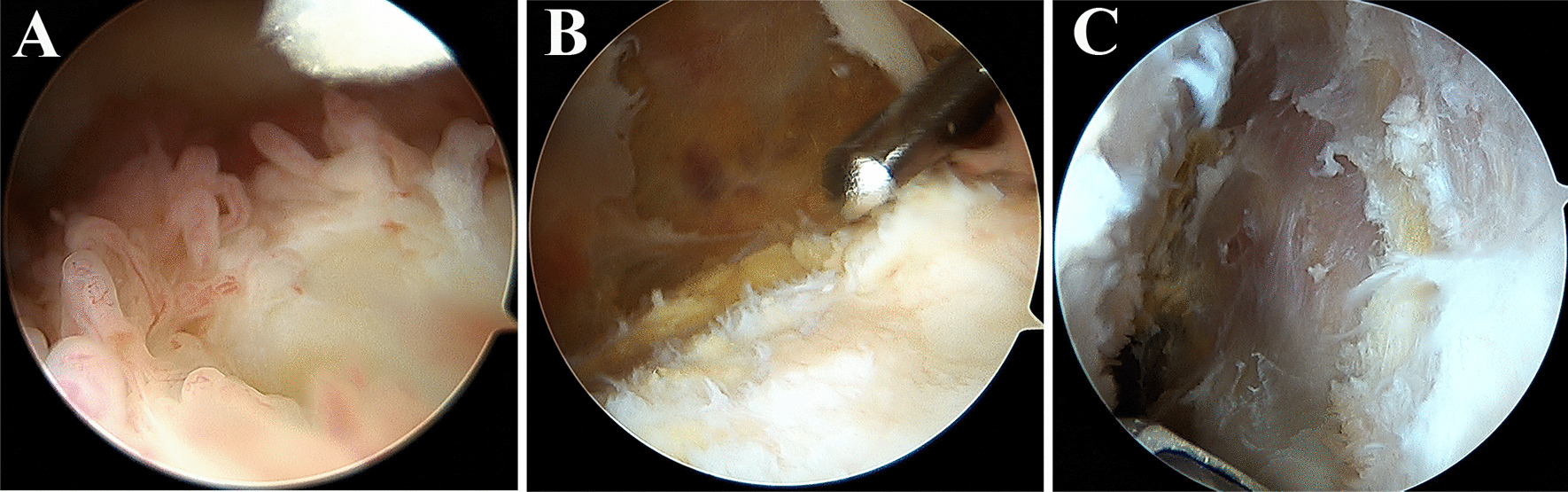
Extensive hyperplasia of hemosiderin rich villous synovium can be seen within the joint cavity under arthroscopy **A**, and **B**, **C** show arthroscopic shaving of the synovial depth requiring excision of the hyperplastic lesion synovium until the fibrous layer of the joint capsule is exposed

The patient was placed in a supine position, and the combined spinal-epidural nerve block (intratracheal general anesthesia for children and patients unable to lumbar puncture), a pneumatic tourniquet on the upper-middle and upper thighs (200mmHg for children, 350mmHg for adults, 90 min), and the normal saline with the specification of 3 L/Bag was suspended at a height of about 1.5 m from the operating table for joint cavity irrigation. The anterolateral approach of the patella is routinely used to examine the suprapatellar capsule, patellofemoral joint, medial groove, medial joint space, intercondylar fossa, lateral joint space, and lateral groove in turn to understand the distribution and proliferation of synovium, as well as ligaments, and semilunar involvement of the plate. After the exploration, an operating instrument was placed in the anteromedial approach, and part of the synovial tissue was clipped with forceps at the typical site of synovial hyperplasia for pathological examination. Subsequent HE staining was performed. Then a planer was used to operate alternately in the anteromedial and anterolateral approach, sequentially removing diseased synovial tissue in the surface of the suprapatellar pouch, medial groove, inferomedial groove and intercondylar groove, lateral groove and inferolateral groove, and medial and lateral meniscal. For diseased synovial tissue in the cruciate ligaments as well as on the medial and lateral patellar surfaces, they also need to be cleared together. The most critical surgical approach is to find the synovial space between the synovial layer and the muscle layer, based on this space, the synovial layer, the sub-synovial layer and the adipose layer were nearly completely peeled off, and the stripping depth reached to the muscle layer. The intraoperative image is shown in Fig. 2. Finally, the joint cavity was rinsed thoroughly with a large amount of normal saline, the intra-articular saline was sucked up before suturing the surgical port, and injecting 5ml of local anesthetic along with 40 mg of triamcinolone acetonide acetate into the joint cavity from the outer edge of the patella with a syringe for postoperative analgesia and avoiding joint adhesion. There was 1 built-in drainage tube for closed negative pressure drainage. Appropriate pressure dressing with cotton pads and elastic bandages was applied to the affected limb.

### Histological staining

Synovial tissues were harvested and fixed with 4% paraformaldehyde for 2 h. They were subsequently dehydrated and embedded in paraffin. Paraffin sections were made to a thickness of 5 μm. Conventional HE staining was subsequently performed, and the stained sections were scanned using a multifunctional digital pathology scanner (Aperio VERSA 8). Subsequently, image information was acquired.

### Post-operative management and rehabilitation exercises

After the operation, symptomatic treatments such as ice compress, analgesia, stomach protection, and appropriate dehydration were given. Isometric contractions and quadriceps straight leg raises were performed after the patient was awake. After 2 days, the drainage tube was removed depending on the amount of drainage, the dressing was changed every other day, and the wound was properly pressurized and bandaged; after the drainage tube was removed, active knee flexion and extension exercises and continuous passive training (1/day, 30 min/time) were performed, and active and passive training was required 1 week after surgery including bending the knee to ≥ 120°. None of the 19 cases showed excessive joint effusion after the operation, and the patients were instructed to avoid strenuous activities within half a year after discharge and to perform daily straight-leg raising exercises.

### Efficacy evaluation criteria

The main evaluation index was the recurrence rate at the last follow-up, and the secondary evaluation indexes were the VAS and the TLS [8] at the last follow-up compared with the preoperative data.

### Statistical analysis

SPSS 25.0 software package was used for data processing. The VAS and TLS were used for paired data t-test before knee arthroscopy and during the return visit. All test data were expressed as (Mean ± SD), and the test level was set as α = 0.05.

## Result

### The histological structure of the synovium

**Fig. 3:**
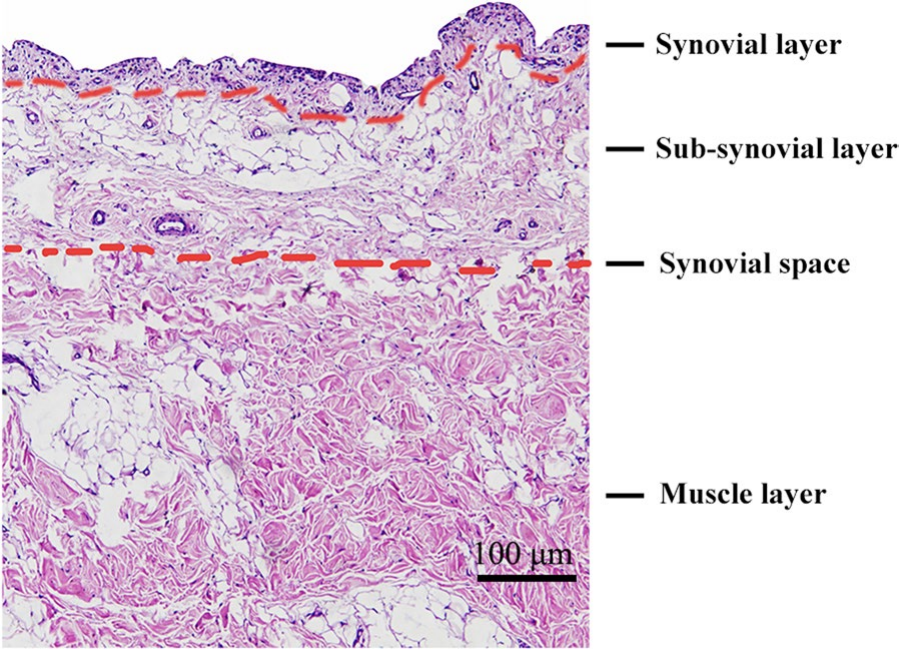
The tissue structure of the synovium. This synovial space is the peeling interface when the arthroscopic synovial peeling method is performed

The synovial space between the synovial layer and the muscle layer was found, based on this space, the synovial layer, the sub-synovial layer and the adipose layer were nearly completely peeled off. The histological structure of the synovium is shown in Fig. 3.

### Recurrence rates

**Fig. 4:**
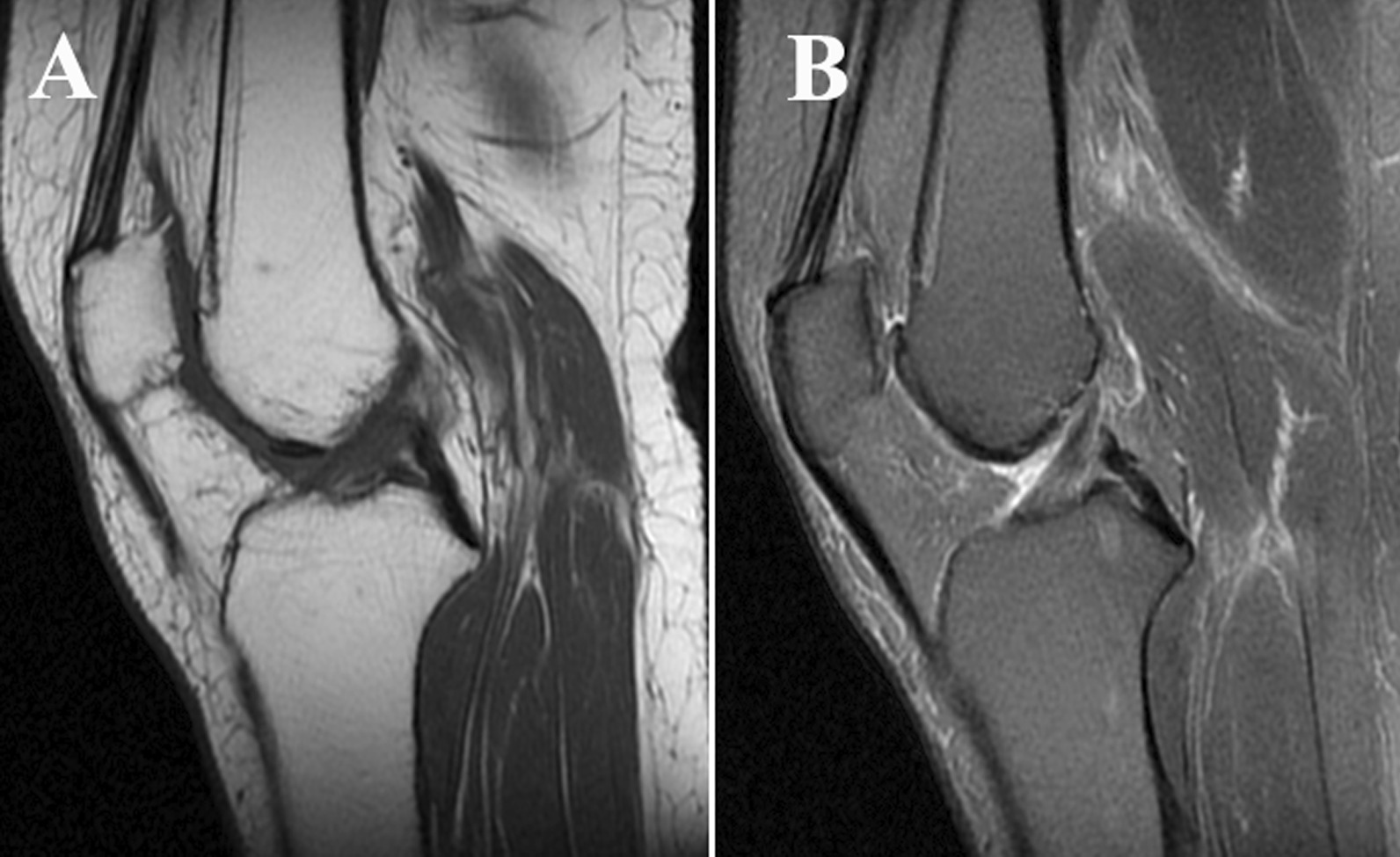
Postoperative MRI showing clear knee joint space and no synovial thickening. **A**. T1WI, **B**. T2WI

Follow-up was obtained postoperatively in all cases, the wounds had all healed by stage I at the 1-month postoperative follow-up, and the joint mobility, joint effusion, and pain showed significant improvement postoperatively than they did preoperatively. Among the treated patients, 2 of them suffered from postoperative recurrence. The criteria for the diagnosis of postoperative recurrence is PVNS confirmed by pathological examination. The two cases suffered recurrence at 7 and 10 months after operation, respectively (both were treated by secondary surgery and showed no recurrence manifestations in subsequent follow-ups), giving a recurrence rate of 10.5%, which is much smaller than reported in the literature [9]. MRI examination of non-recurred case, as shown in Fig. 4.

### Functional outcome

**Fig. 5:**
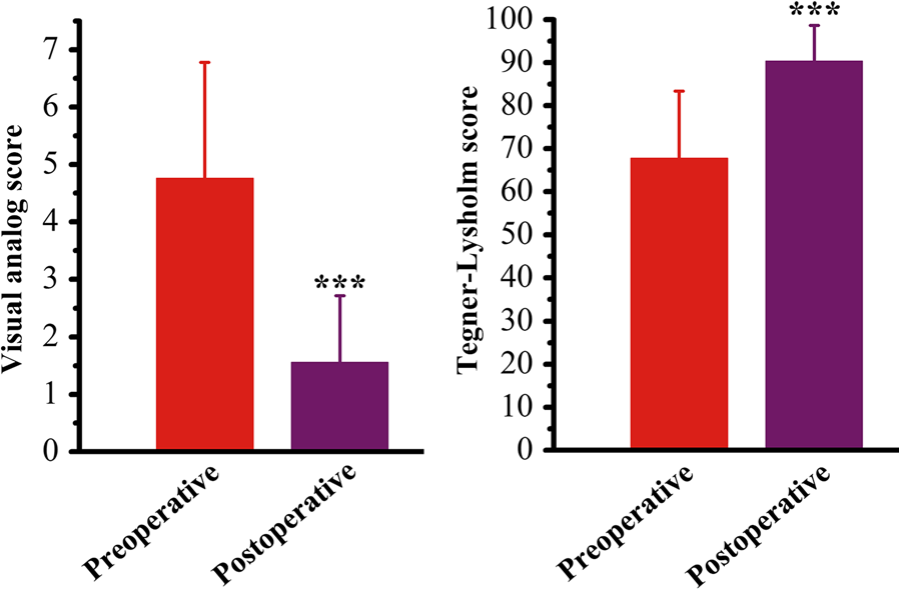
Statistical results of VAS and TLS. ***Represents compared with the preoperation group, P less than 0.001

As indicated in Fig. 5, the VAS reduced from 4.76 ± 2.02 preoperational to 1.56 ± 1.15 at the last follow-up (19th August, 2021) (P < 0.001), and the TLS improved from 67.76 ± 15.64 to 90.32 ± 8.32 at the last follow-up (19th August, 2021) (P < 0.001). The functional outcome calculated from all 19 cases. These score values all indicated a good functional prognosis.

## Discussion

There are many factors contributing to the recurrence of PVNS. The prevailing view currently holds that incomplete clearance of diseased synovial tissue is the underlying cause of PVNS recurrence [10]. At present, the reported methods are only to resect the pathological synovial tissue. There are various ways of resection, which often cannot guarantee complete resection. For the first time, we clarified how to nearly completely remove the pathological synovial tissue from the perspective of tissue anatomy.

Based on the histological characteristics of synovial tissue, the method of peel is used, resulting in not only decreased bleeding but also nearly complete decortication of lesion tissue, which considerably minimizes recurrence and is suitable for clinical promotion. PVNS, particularly diffuse PVNS, is distinguished by its proclivity for recurrence [11–13]. Mollon et al. [14] showed an overall incidence of 21.8% after surgical resection of PVNS. Patel et al. [15] found a high recurrence rate of 47.6% after diffuse PVNS surgery with the use of arthroscopic procedures through a retrospective analysis of 214 patients. Hans Roland Dürr et al. [16] counted a 24% recurrence rate after surgical resection of PVNS, and there was no significant difference in the recurrence rate after surgical synovectomy of PVNS with or without adjunctive radiological synovioplasty (RSO). QinWei Guo et al. [11] demonstrated a higher recurrence rate in PNVS with lesion metastasis to the extra articular site compared to common PNVS. Studies have indicated that the preoperative neutrophil to lymphocyte ratio is valuable in predicting recurrence of PVNS of the knee [17]. Ogilvie Harris et al. [18] manifested that whether complete resection of diseased synovial tissue is the key factor affecting whether PVNS recur. Our team’s innovative approach to synovial peel was designed to overcome the current problem of incomplete synovial resection. According to statistics, our postoperative recurrence rate of PVNS was lowered to 10.5%, which is significantly lower than the more than 20% currently reported.

We found that the patients’ pain symptoms and joint function scores were significantly improved after treatment by ATSP through the postoperative follow-up statistics. The postoperative recovery of joint function was greatly improved, which has a close relationship with the less traumatic nature of arthroscopic surgery, as patients frequently appear with joint function issues following open surgery [19, 20]. Second, the entire treatment course was performed without radiological or chemical synostosis. Particularly radioisotope synovectomy must be performed by experienced professionals because of its potential for significant complications [21].

We treated diffuse PVNS starting from the anatomical characteristics of the synovium, and the study had certain limitations. First, the sample size of the present study was small, which was related to the characteristics of the low incidence of PVNS. Second, individual patient follow-up was relatively short. As is known to all, the longer the follow-up, the more reliable the data obtained. Carl Ferdinand Capellen et al. [22] suggested that 18% of patients recurred within 18 months, and more than 90% of patients recurred within the first three years. This seems to illustrate that the recurrence of diffuse PVNS is mainly within three years after surgery.

## Conclusion

An arthroscopic synovectomy is the main method for the treatment of PVNS. We have greatly reduced the recurrence rate of patients with diffuse lesions by total synovial peel. Moreover, the knee function recovered significantly after the operation, with less trauma and rapid recovery. Therefore, the preliminarily findings suggest that ATSP in the treatment of PVNS is worthy of clinical promotion.

## Data Availability

The datasets used and/or analyzed during the current study are available from the corresponding author on reasonable request.

## Abbreviations

PVNS: Pigmented villonodular synovitis
ATSP: Arthroscopic total synovial peel
VAS: Visual analog score
TLS: Tegner-Lysholm score
RSO: Radiological synovioplasty
